# Vasculotide reduces pulmonary hyperpermeability in experimental pneumococcal pneumonia

**DOI:** 10.1186/s13054-017-1851-6

**Published:** 2017-11-13

**Authors:** Birgitt Gutbier, Xiaohui Jiang, Kristina Dietert, Carolin Ehrler, Jasmin Lienau, Paul Van Slyke, Harold Kim, Van C. Hoang, Jason T. Maynes, Daniel J. Dumont, Achim D. Gruber, Norbert Weissmann, Timothy J. Mitchell, Norbert Suttorp, Martin Witzenrath

**Affiliations:** 1 Charité – Universitätsmedizin Berlin, corporate member of Freie Universität Berlin, Humboldt-Universität zu Berlin, and Berlin Institute of Health, Department of Infectious Diseases and Pulmonary Medicine, Charitéplatz 1, 10117 Berlin, Germany; 2 Institute of Veterinary Pathology, Freie Universität Berlin, Robert-von-Ostertag-Strasse 15, 14163 Berlin, Germany; 3 Vasomune Therapeutics, 661 University Avenue, Suite 465, Toronto, ON M5G 1M1 Canada; 4 Department of Anesthesia and Pain Medicine, Hospital for Sick Children, Toronto, ON M5G 1X8 Canada; 5 Departments of Anesthesia and Biochemistry, University of Toronto, Toronto, ON M5S 2J7 Canada; 6 Sunnybrook Research Institute, Sunnybrook Health Sciences Centre, 2075 Bayview Avenue, Toronto, ON M4N 3M5 Canada; 7 Excellence Cluster Cardio-Pulmonary System, University of Giessen and Marburg Lung Center (UGMLC), German Center for Lung Research (DZL), Justus-Liebig-University, Giessen, 35392 Germany; 8 Institute of Microbiology and Infection, College of Medical and Dental Sciences, University of Birmingham, Edgbaston, Birmingham, B15 2TT UK

**Keywords:** Pneumococcal pneumonia, Pneumolysin, Angiopoietins, Acute lung injury, Vasculotide

## Abstract

**Background:**

Community-acquired pneumonia (CAP) is a significant cause of morbidity and mortality worldwide. Despite effective antimicrobial therapy, CAP can induce pulmonary endothelial hyperpermeability resulting in life-threatening lung failure due to an exaggerated host-pathogen interaction. Treatment of acute lung injury is mainly supportive because key elements of inflammation-induced barrier disruption remain undetermined. Angiopoietin-1 (Ang-1)-mediated Tie2 activation reduces, and the Ang-1 antagonist Ang-2 increases, inflammation and endothelial permeability in sepsis. Vasculotide (VT) is a polyethylene glycol-clustered Tie2-binding peptide that mimics the actions of Ang-1. The aim of our study was to experimentally test whether VT is capable of diminishing pneumonia-induced lung injury.

**Methods:**

VT binding and phosphorylation of Tie2 were analyzed using tryptophan fluorescence spectroscopy and phospho-Tie-2 enzyme-linked immunosorbent assay. Human and murine lung endothelial cells were investigated by immunofluorescence staining and electric cell-substrate impedance sensing. Pulmonary hyperpermeability was quantified in VT-pretreated, isolated, perfused, and ventilated mouse lungs stimulated with the pneumococcal exotoxin pneumolysin (PLY). Furthermore, *Streptococcus pneumoniae*-infected mice were therapeutically treated with VT.

**Results:**

VT showed dose-dependent binding and phosphorylation of Tie2. Pretreatment with VT protected lung endothelial cell monolayers from PLY-induced disruption. In isolated mouse lungs, VT decreased PLY-induced pulmonary permeability. Likewise, therapeutic treatment with VT of *S. pneumoniae*-infected mice significantly reduced pneumonia-induced hyperpermeability. However, effects by VT on the pulmonary or systemic inflammatory response were not observed.

**Conclusions:**

VT promoted pulmonary endothelial stability and reduced lung permeability in different models of pneumococcal pneumonia. Thus, VT may provide a novel therapeutic perspective for reduction of permeability in pneumococcal pneumonia-induced lung injury.

**Electronic supplementary material:**

The online version of this article (doi:10.1186/s13054-017-1851-6) contains supplementary material, which is available to authorized users.

## Background

Community-acquired pneumonia (CAP) is the most common serious infectious disease worldwide [1–4]. *Streptococcus pneumoniae* is the most prevalent causal pathogen identified in CAP [3, 5]. Reaching more than 10%, the lethality of hospitalized patients with pneumonia is relatively high [6–8]. Despite effective antibiotic therapy, pathogen-host interaction in pneumonia may increase pulmonary endothelial permeability, leading to the formation of interstitial and alveolar protein-rich edema, and finally to life-threatening lung failure [9]. Mainly supportive therapies are available for acute lung injury, but specific treatments to protect the pulmonary barrier function are lacking [10–12]. Thus, there is a need for novel therapeutic strategies in addition to antibiotics to avoid pneumonia-induced acute lung injury.

Angiopoietins (Ang-1 to Ang-4) are ligands for the receptor tyrosine kinase Tie2 [13]. Ang-1 and Ang-2, currently the best described angiopoietins, are well-known contributors to angiogenesis [14] and regulators of inflammation and vascular leakage [15–18]. Constitutively released Ang-1 activates the Tie2 receptor, resulting in reduction of inflammation and apoptosis as well as stabilization of endothelial barrier function [18–21]. In contrast, Ang-2 supports inflammation and permeability by acting as an Ang-1 antagonist at Tie2 [15, 22].

Vasculotide (VT) is a synthetic peptide clustered with polyethylene glycol [23, 24]. VT was shown to protect endothelial cells from lipopolysaccharide (LPS)-induced disruption and to prevent endotoxemia-induced lung vascular leakage [25], diminish barrier disruption in experimental sepsis [26] and acute kidney injury [27], and increase the survival of mice with influenza pneumonia [28]. However, its potential to support pulmonary barrier function in severe pneumococcal pneumonia has not been investigated so far.

In order to characterize effects of VT on pneumonia-induced acute lung injury, we used several approaches with differing levels of complexity. In vitro human and murine pulmonary endothelial cell monolayers were pretreated with VT and stimulated with pneumolysin (PLY), an exotoxin of *S. pneumoniae* [29, 30]. Likewise, ex vivo perfused and ventilated mouse lungs were treated with VT and stimulated with PLY. Finally, VT was applied in vivo to treat mice with severe pneumococcal pneumonia therapeutically. Some of the results presented here were previously reported in the form of abstracts [31, 32].

## Methods

### Tryptophan fluorescence spectroscopy

Binding studies were carried out in 96 well plates (Brand-plates pureGrade reference 781607; BRAND GMBH + CO KG, Wertheim, Germany). Mouse Tie2Fc receptor and human immunoglobulin G (IgG) Fc were obtained from R&D Systems (Minneapolis, MN, USA). Dilutions were prepared in HyClone™ Dulbecco’s PBS (GE Healthcare Life Sciences, Mississauga, ON, Canada). The saturation binding curve was prepared with all ligand dilutions and receptors as 2× concentrations. Equal volumes of ligand and receptor were added to generate a final 1× concentration (500 nM Tie2Fc final concentration). Spectroscopic studies were performed on a SpectraMax M2 fluorometer (Molecular Devices, Sunnyvale, CA, USA). Measurements were taken at an excitation wavelength of 295 nm. Emission scans were set from 360 to 450 nm.

### Tie2 phosphorylation analysis in vitro

C57BL/6 mouse primary lung microvascular endothelial cells (Cell Biologics, Chicago, IL, USA) were grown in endothelial cell medium with a kit (Cell Biologics) in 20% O_2_/5% CO_2_ in a 37 °C humidified chamber and split 1:2 once reaching 90–95% confluency. To examine the activation of Tie2 by VT in mouse primary lung microvascular endothelial cells, 95–100% confluent cells were stimulated with VT (1, 10, 100, and 1000 ng/ml) in serum-containing media for 15 minutes. Human Ang-1 (R&D Systems) was used as a positive control. VT was synthesized, purified, and validated as described before [24, 26, 33]. Cell lysates were collected using lysis buffer recommended in the mouse phospho-Tie-2 enzyme-linked immunosorbent assay (ELISA) kit (R&D Systems); tyrosine-phosphorylated Tie2 and total Tie2 were measured using ELISA kits (mouse phospho-Tie2 and total-Tie2 ELISA kits; R&D Systems). Three to six independent experiments were analyzed.

### Immunofluorescence staining of human pulmonary microvascular endothelial cells

Human pulmonary microvascular endothelial cells (hPMVEC; PromoCell, Heidelberg, Germany), used at passages 4–6, were grown to confluence on glass slides, incubated with VT (300 ng/ml) or sterile PBS as a control for 90 minutes and then stimulated with LPS (0.1 μg/ml) from *Escherichia coli* (Sigma-Aldrich, Steinheim, Germany), PLY (0.25, 0.5, 0.75 μg/ml) [34], or PBS. After 30 minutes, cells were fixed in 3% formalin, and the tight junction protein vascular endothelial (VE)-cadherin (1:400, goat antihuman VE-cadherin; Santa Cruz Biotechnology, Santa Cruz, CA, USA; secondary antibody 1:8000, Alexa Fluor 488 F(ab′)_2_ rabbit antigoat IgG (H + L), Invitrogen, Darmstadt, Germany) and actin fibers (1:2000; phalloidin Alexa 546-labeled; Invitrogen) were stained to evaluate cell morphology [35]. 4′,6-diamidino-2-phenylindole staining (Sigma-Aldrich) was used to visualize cell nuclei. The immunofluorescence of the cells was analyzed by confocal microscopy using an LSM 780 microscope with ZEN 2011 software (objective: Plan Apochromat × 63/1.40 oil; Carl Zeiss Microscopy, Jena, Germany).

### Animals

For all experiments, female C57BL/6 N mice (8 to 10 weeks old, weighing 18 to 20 g; Charles River Laboratories, Sulzfeld, Germany) were used. Animals were housed under specific pathogen-free conditions under a 12–h/12-h light/dark cycle with free access to food and water. Animal housing and experimental procedures complied with the Federation of European Laboratory Animal Science Associations (FELASA) guidelines and recommendations for the care and use of laboratory animals.

### Transcellular electrical resistance of murine lung endothelial cells

Murine lung endothelial cells (mLEC) were isolated and purified according to protocols published previously [36, 37]. Briefly, mice were killed by an overdose of ketamine/xylazine. Lungs of four mice were pooled, minced, and digested by dispase (20 ml, 5 U/ml; BD Biosciences, Heidelberg, Germany) and DNase (20 ml, 0.5 mg/ml; AppliChem GmbH, Darmstadt, Germany). The homogenate was filtered, collected by centrifugation (300 × *g*), and washed twice, and the resulting cell suspension was incubated with antimouse VE-cadherin antibody (rat antimouse CD 144; BD Pharmingen, San Diego, CA, USA)-coated magnetic beads (sheep antirat IgG DynaBeads; Invitrogen GmbH, Karlsruhe, Germany) for positive isolation with the DynaMag-Spin magnet system (Invitrogen). The isolated cells were resuspended in Endothelial Cell Growth Medium MV2 (PromoCell), seeded onto fibronectin-coated (Sigma-Aldrich) culture dishes (diameter 35 mm), and grown to a confluent monolayer in 3–4 days. Then, cells were split after trypsinization [37], seeded and grown to confluence on fibronectin-coated evaporated gold microelectrodes (eight-well array with ten electrodes per well; ibidi GmbH, Martinsried, Germany) connected to an electric cell-substrate impedance sensing (ECIS) system (Applied Biophysics, Troy, NY, USA) [38] for continuous monitoring of transcellular electrical resistance (TER). Cells were pretreated with VT (2, 10, and 50 ng/ml) or PBS for 30 minutes and then stimulated with PLY (0.75 μg/ml) or PBS for 3 h.

### Isolated perfused mouse lung

Mouse lungs were prepared as described previously [39]. Briefly, anesthetized mice were tracheotomized and ventilated. After sternotomy and cannulation of the left atrium and pulmonary artery, lungs were perfused with 37 °C sterile Krebs-Henseleit hydroxyethyl-amylopectin buffer (1 ml/minute) and ventilated by negative pressure (P_exp_ −4.5, P_ins_ −9.0 cmH_2_O) in a humidified chamber. After a 20-minute steady state, continuous infusion of VT (50 or 100 ng/ml) or PBS was commenced. Five minutes later, human serum albumin (HSA) was admixed to the perfusate (0.04%) for permeability quantification. Ten minutes thereafter, 1.4 μg/ml PLY was infused into the pulmonary artery for 1 minute. Bronchoalveolar lavage (BAL) was performed 30 minutes after PLY application, and HSA concentration was measured in bronchoalveolar fluid (BALF) [35, 40].

### Murine pneumonia and vasculotide therapy

Mice were anesthetized with ketamine (80 mg/kg) and xylazine (25 mg/kg) and transnasally inoculated with 5 × 10^6^ colony-forming units (CFU) of *S. pneumoniae* (NCTC7978) in 20 μl of sterile PBS as described before [35]. Sham-infected control mice received 20 μl of sterile PBS. After 22 h, 34 h, and 46 h, body weight and rectal temperature were measured, and different dosages of VT (100, 200, or 500 ng in 100 μl of PBS) or PBS were intravenously injected into the tail vein. Twenty-four or forty-eight hours postinfection (p.i.), mice were anesthetized (160 mg/kg ketamine and 75 mg/kg xylazine) and exsanguinated. The experimental design is shown in Additional file 1: Figure S1. Subsequently, lungs were flushed, and BAL was performed. To determine permeability, HSA (1 mg/75 μl) was intravenously infused 1 h before lung preparation, and the BALF/plasma ratio was calculated as described elsewhere [35]. BALF leukocytes were differentiated by fluorescence-activated cell sorting (FACSCanto II; BD Biosciences) using forward- vs. side-scatter characteristics and peridinin chlorophyll protein complex-CD45 (clone 30-F11; BD Biosciences), V450-Ly6G (clone 1A8; BD Biosciences), and phycoerythrin-F4/80 (clone BM8; eBioscience, Vienna, Austria) staining. Blood leukocytes were quantified using a scil Vet ABC Hematology Analyzer (scil animal care company GmbH, Viernheim, Germany). Cytokines from BALF were measured with a multiplex assay (ProcartaPlex; eBioscience), and plasma levels of interleukin (IL)-6 (BD Biosciences) and KC (keratinocyte chemoattractant; R&D Systems) were quantified by ELISA.

Identical experiments were performed separately for histological analyses of the lungs. Mice were anesthetized 24 h or 48 h p.i. (160 mg/kg ketamine and 75 mg/kg xylazine), heparinized, and exsanguinated. After ligation of the trachea, mouse lungs were removed and immediately immersion-fixed in 4% buffered formalin, embedded in paraffin, cut into 2-μm-thick sections, and stained with hematoxylin and eosin as described elsewhere [41, 42]. The degree of edema formation was assessed semiquantitatively (0 = no edema, 1 = minimal edema, 2 = mild edema, 3 = moderate edema, 4 = severe edema). Three evenly distributed sections per lung were microscopically evaluated to assess edema formation.

### Statistical analysis

**Fig. 1:**
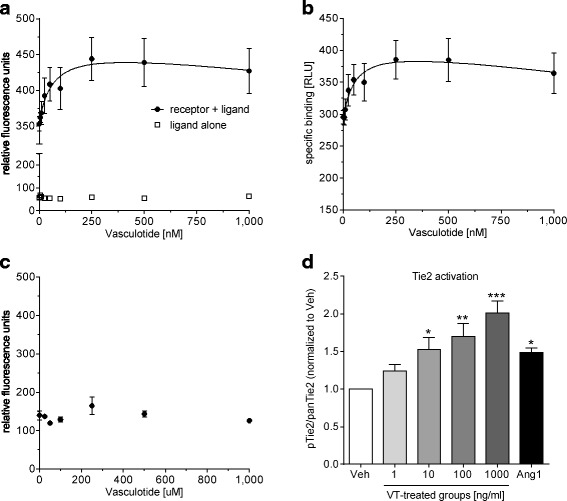
Vasculotide (VT) binds and phosphorylates mouse Tie2. **a** Fluorescence intensity with the incubation of mouse Tie2Fc receptor and VT (*closed circles*) or VT alone (*open squares*). **b** Relative light units (RLU) of the specific binding of VT to Tie2Fc, which were corrected to the baseline RLU values of VT alone, are shown. The dissociation constant of the normalized specific binding of VT to mouse Tie2Fc was 34.95 nM (95% CI 2.92–66.97 nM). Values were normalized to the bottom plateau (RLU value of 0 nM VT) and the top plateau at saturation. **c** No saturation of binding is observed with human immunoglobulin G Fc and VT. **d** Mouse primary lung microvascular endothelial cells were stimulated with 1, 10, 100, and 1000 ng/ml VT and 800 ng/ml human angiopoietin-1 and normalized to the vehicle-treated group. Data represent mean ± SEM (*n* = 3–6). *p < 0.05, **p < 0.01, ***p < 0.001 by one-way analysis of variance (Dunnett’s post hoc test)

Saturation binding data (Fig. 1a–c) are presented as mean ± SEM and were calculated using normalized one-site specific binding and nonlinear regression. For the Tie2 phosphorylation analysis (Fig. 1d), one-way analysis of variance (with Dunnett’s post hoc multiple comparisons test) was used to compare treatment groups with the vehicle-treated group. All other experimental data are expressed as mean, mean + SEM, or mean ± SEM. Groups were compared using the Mann-Whitney *U* test followed by the Bonferroni-Holm correction, where indicated. *p* Values < 0.05 were considered significant. Analyses were performed using Prism version 6.00 software (GraphPad Software, La Jolla, CA, USA).

## Results

### Vasculotide binds and phosphorylates mouse Tie2

Using tryptophan fluorescence spectroscopy, a dose-dependent increase in intrinsic fluorescence intensity was observed with the incubation of recombinant mouse Tie2Fc receptor and VT (Fig. 1a and b). The fluorescence intensity was found to be saturable. The dissociation constant of the normalized specific binding of VT to mouse Tie2Fc was 34.95 nM (95% CI 2.92–66.97 nM). For this calculation, the relative light units (RLU) were normalized to the bottom plateau (RLU value of 0 nM VT) and the top plateau at saturation. Significantly, no dose-dependent increase in fluorescence intensity was observed with recombinant human IgGFc and VT (Fig. 1c). Furthermore, stimulation of mouse primary lung microvascular endothelial cells with VT resulted in Tie2 phosphorylation in a dose-dependent manner (Fig. 1d).

### Vasculotide reduced pneumolysin-evoked barrier disruption of lung endothelial cells in vitro

**Fig. 2:**
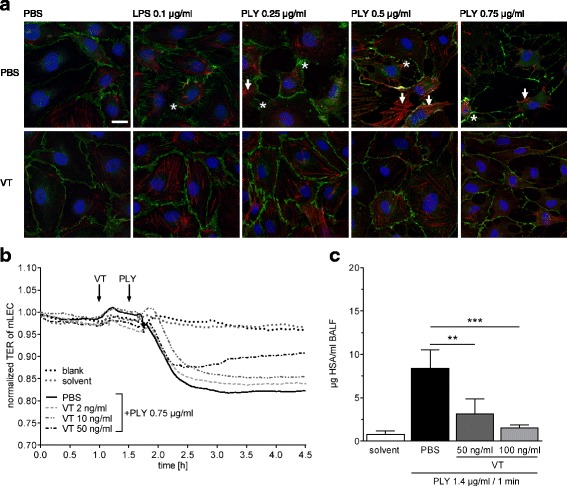
Vasculotide (VT) stabilized endothelial barrier function in vitro and ex vivo. **a** Confluent human pulmonary microvascular endothelial cells (hPMVEC) were preincubated for 90 minutes with 300 ng/ml VT or solvent (PBS) and stimulated with lipopolysaccharide (LPS; 0.1 μg/ml), pneumolysin (PLY; 0.25, 0.5, or 0.75 μg/ml), or PBS for 30 minutes. Cells were fixed and vascular endothelial (VE)-cadherin (*green*), actin fibers (*red*), and cell nuclei (*blue*) were stained for immunofluorescence microscopy. Unstimulated, PBS-treated, or VT-treated cells showed intact monolayers with tight intercellular contacts and thin actin fibers (*red*). In PBS-pretreated groups, the integrity between cell contacts was disrupted, and hPMVEC showed gap formation (*asterisks*) and stress fibers (*arrows*) after LPS or PLY stimulation (*upper row*). Pretreatment with VT stabilized the cell monolayer, avoided gap formation, and reduced stress fibers and cell loss from culture dishes (*lower row*). Representative images of three independent experiments are shown for every group. **a** Bar = 20 μm (valid for all photomicrographs). **b** Transcellular electrical resistance (TER) of isolated murine lung endothelial cell (mLEC) monolayers was continuously monitored. mLEC were pretreated for 30 minutes with VT (2, 10, or 50 ng/ml) or PBS and then stimulated with pneumolysin (PLY; 0.75 μg/ml). PLY stimulation decreased TER of mLEC monolayers, displaying loss of endothelial integrity (*solid curve*). Preincubation with VT attenuated the PLY-induced TER decrease in a dose-dependent manner (*dashed* and *dash-dotted curves*). **c** Ex vivo perfused and ventilated mouse lungs were pretreated with VT (50 ng/ml or 100 ng/ml) or PBS 15 minutes before PLY stimulation (1.4 μg/ml). After 30 minutes, lung vascular permeability was assessed by quantifying the concentration of continuously infused human serum albumin (HSA) in bronchoalveolar lavage fluid (BALF). Treatment with VT significantly decreased hyperpermeability of mouse lungs as compared with PBS treatment. Values are given as mean (C; *n* = 4–7) or mean + SEM (D; *n* = 6–8). **p < 0.01, ***p < 0.001 between indicated groups

In a first step, effects of VT on PLY-evoked barrier failure [35] in hPMVEC were analyzed. VT had been shown to protect against LPS-induced cell damage [25]. For comparability, we first stimulated cell monolayers with LPS. In the absence of LPS or PLY, VE-cadherin staining (Fig. 2a) revealed an intact monolayer with tight cell contacts and thin actin fibers, and no effect of VT pretreatment was observed As expected, PBS-pretreated hPMVEC showed gap formation between cells and generation of stress fibers after LPS (0.1 μg/ml) or PLY (0.25, 0.5 μg/ml) challenge, as well as strong cell retraction and loss of cells after stimulation with 0.75 μg/ml PLY (Fig. 2a, *upper row*). Pretreatment with VT (300 ng/ml) stabilized cell monolayers, protected from gap formation, and reduced stress fibers as well as cell loss (Fig. 2a, *lower row*).

In additional ECIS experiments, PLY stimulation evoked a decrease in TER of mLEC monolayers, displaying loss of endothelial integrity (Fig. 2b, *solid curve*). Incubation of confluent mLEC monolayers with VT attenuated the PLY-induced TER decrease in a dose-dependent manner (Fig. 2b, *dashed*/*dash-dotted curves*).

### Vasculotide reduced pneumolysin-evoked permeability in isolated perfused and ventilated mouse lungs

PLY stimulation increased lung permeability 30 minutes after application (Fig. 2c) as described before [35]. Treatment with VT 15 minutes before PLY application dose-dependently decreased hyperpermeability of mouse lungs (Fig. 2c).

### Vasculotide therapy protected pulmonary barrier function without affecting immune response in murine pneumococcal pneumonia

**Fig. 3:**
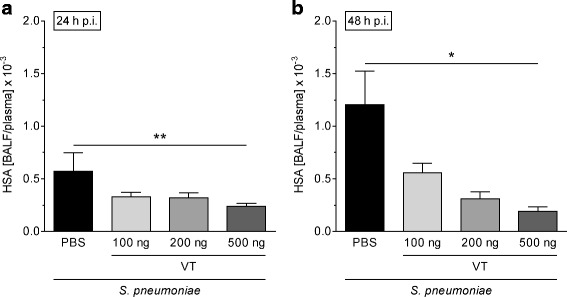
Vasculotide (VT) therapy decreased pulmonary hyperpermeability in *Streptococcus pneumoniae*-infected mice. *S. pneumoniae*-infected mice (5 × 10^6^ colony-forming units/mouse) were intravenously (i.v.) treated with VT (100, 200, or 500 ng) or PBS 22 h, 34 h, and 46 h postinfection (p.i.). Lung preparation and bronchoalveolar lavage were performed 24 h or 48 h p.i. Human serum albumin (HSA) was i.v. administered 23 h or 47 h p.i., and HSA bronchoalveolar lavage fluid (BALF)/plasma ratio was determined by enzyme-linked immunosorbent assay to quantify permeability. At a dose of 500 ng, VT significantly reduced lung hyperpermeability 24 h (**a**) and 48 h (**b**) p.i. compared with the PBS-treated controls. Values are given as mean + SEM (*n* = 5–7). *p < 0.05, **p < 0.01 between indicated groups

**Fig. 4:**
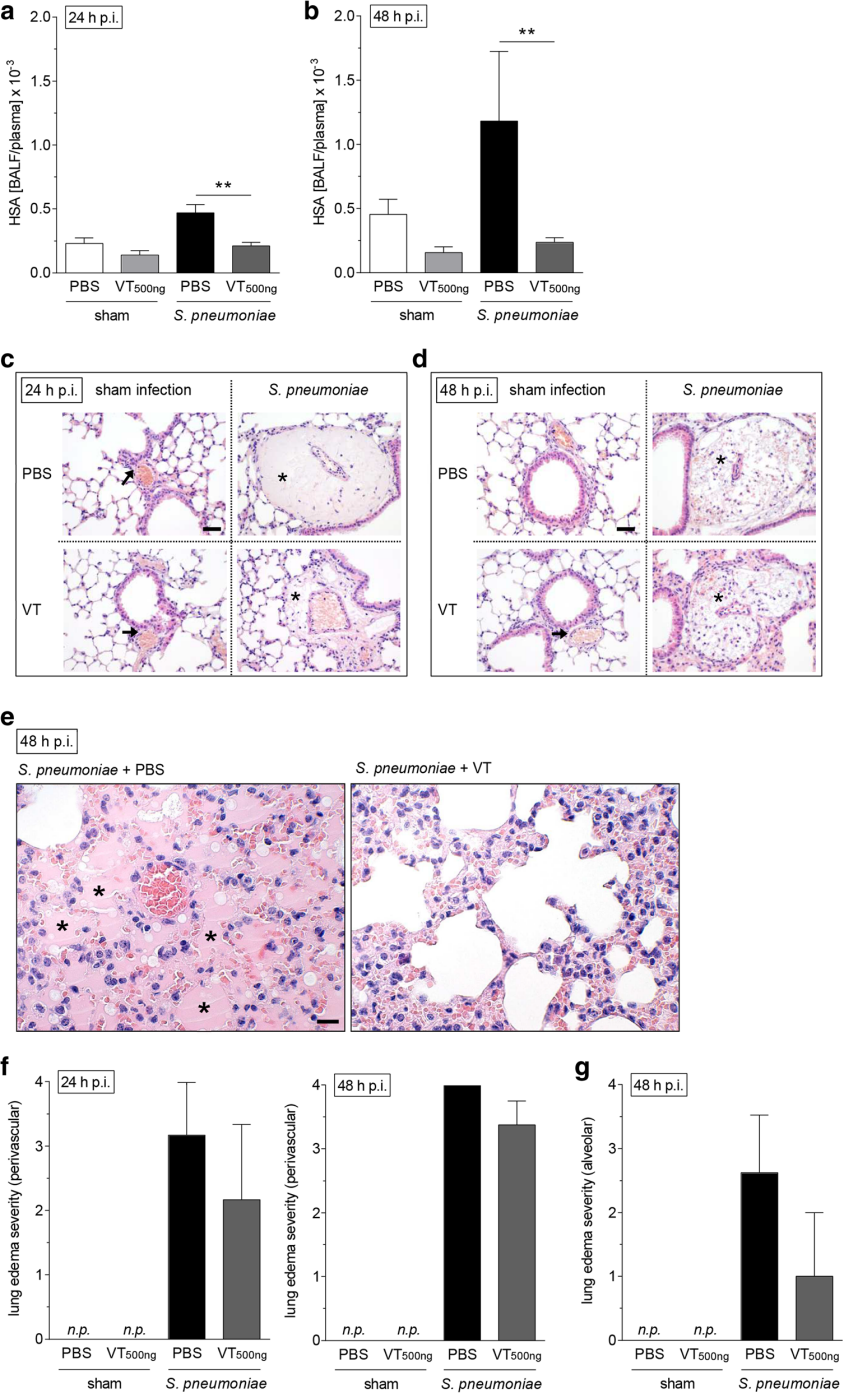
Improvement of lung barrier function by Vasculotide (VT) was associated with diminished edema formation. *Streptococcus pneumoniae* (5 × 10^6^ colony-forming units/mouse) or sham-infected mice were intravenously (i.v.) treated with VT (500 ng) or PBS 22 h, 34 h, and 46 h postinfection (p.i.). Lungs were prepared, and bronchoalveolar lavage was performed 24 h or 48 h p.i. Human serum albumin (HSA) was i.v. administered 23 h or 47 h p.i., and HSA bronchoalveolar lavage fluid (BALF)/plasma ratio was determined by enzyme-linked immunosorbent assay to quantify permeability. For histological analysis, lungs were prepared and fixed 24 h or 48 h p.i. **a** and **b** Lung permeability was decreased 24 h and 48 h p.i. upon VT treatment. **c** Histological analysis revealed that infected and PBS-treated mice showed massive perivascular edema formation 24 h after infection (*asterisks*), which was considerably reduced by VT treatment. **d** At 48 h p.i., the edematous perivascular spaces in the lungs were increasingly infiltrated by neutrophils (*asterisks*), but without differences between the VT-treated and untreated infected groups. In the sham-infected groups, no edema formation or other pathological changes could be seen in the perivascular spaces at both time points (*arrows* in **c** and **d**). **e** PBS-treated mice showed massive alveolar edema formation 48 h after infection (*asterisks*), which was almost completely abolished (three of four mice) by VT treatment. **f** and **g** Semiquantitative determination of perivascular (**f**) and alveolar (**g**) edema formation confirmed these observations. **a** and **b** Values are given as mean + SEM (*n* = 8 for *S. pneumoniae*-infected groups or *n* = 5 for sham-infected groups). **p < 0.01 between indicated groups. **c**, **d**, and **e** Representative images are shown (*n* = 3–4); *n.p.* not present. Bars = 100 μm (**c**, **d**), 50 μm (**e**). **f** and **g** Values are given as mean + SEM (*n* = 3–4)

**Fig. 5:**
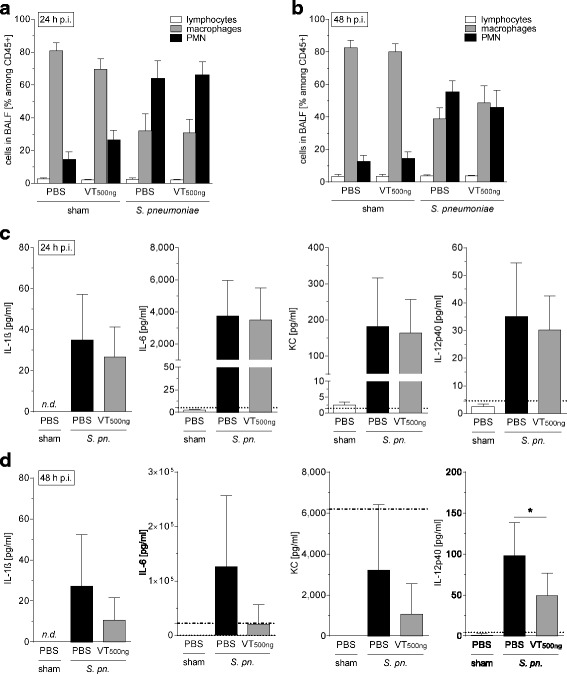
Vasculotide (VT) treatment was not associated with a reduced pulmonary immune response. *Streptococcus pneumoniae* (5 × 10^6^ colony-forming units/mouse) or sham-infected mice were intravenously (i.v.) treated with VT (500 ng) or PBS 22 h, 34 h, and 46 h postinfection (p.i.). Lungs were prepared, and bronchoalveolar lavage was performed 24 h or 48 h p.i. Immune cells in bronchoalveolar lavage fluid (BALF) were differentiated by fluorescence-activated cell sorting, and BALF cytokines were quantified. Pulmonary leukocyte recruitment (**a**, **b**) and BALF cytokine concentrations (**c**, **d**) were not significantly reduced (except interleukin [IL]-12p40) by VT 24 h (**a**, **c**) and 48 h (**b**, **d**) after infection. Values are given as mean + SEM (*n* = 8 for *S. pneumoniae*-infected groups or *n* = 5 for sham-infected groups). *Dotted lines* indicate lower detection limits, and *dash-dotted lines* indicate upper detection limits, of the cytokine assay. *p < 0.05 between indicated groups. *nd* not detectable, *PMN* Polymorphonuclear cells, *S. pn. Streptococcus pneumoniae*

**Fig. 6:**
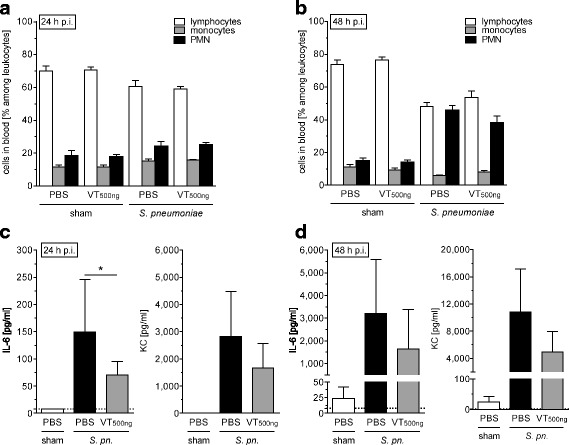
Vasculotide therapy (VT) did not relevantly alter the systemic inflammatory response in *Streptococcus pneumoniae*-infected mice. *S. pneumoniae* (5 × 10^6^ colony-forming units/mouse) or sham-infected mice were intravenously (i.v.) treated with VT (500 ng) or PBS 22 h, 34 h, and 46 h postinfection (p.i.). Mice were exsanguinated, and their lungs were prepared 24 h or 48 h p.i. Blood cells were differentiated, and plasma levels of interleukin (IL)-6 and KC were quantified. Subgroups of blood leukocytes did not differ between VT-treated and solvent (PBS)-treated mice with pneumonia 24 h (**a**) and 48 h (**b**) p.i. IL-6 plasma levels were significantly reduced after VT treatment 24 h p.i. (**c**) but did not differ between infected groups at 48 h p.i. (**d**). Values are given as mean + SEM (*n* = 8 for *S. pneumoniae*-infected groups or *n* = 5 for sham-infected groups). *p < 0.05 between indicated groups. *PMN* Polymorphonuclear cells, *S. pn. Streptococcus pneumoniae*

Mice infected with *S. pneumoniae* were treated with VT when pneumonia was already established (starting point 22 h p.i.) [35, 43]. Body weight and temperature were not affected by VT therapy compared with infected, PBS-treated mice (Additional file 1: Figure S2A and B). However, VT reduced lung hyperpermeability in a dose-dependent fashion, with the highest dosage of VT (500 ng) exerting a significant permeability reduction at 24 h and 48 h p.i. compared with PBS treatment (Fig 3a and b). In a further set of experiments in which mice were treated with the highest dosage of VT and with a focus on the immune response after VT treatment, the decreased pulmonary barrier disruption at 24 h and 48 h after infection was confirmed (Fig. 4a and b). Histological analysis of lung tissue revealed reduced lung injury of infected, VT-treated animals at 24 h and 48 h after infection compared with infected, PBS-treated mice (Additional file 1: Figure S3). Furthermore, infected, PBS-treated mice showed massive perivascular edema formation 24 h after infection, which was considerably reduced by VT treatment (Fig. 4c and f). At 48 h p.i., the edematous perivascular spaces in the lungs were increasingly infiltrated by neutrophils, without differences between the VT-treated and untreated groups. However, VT-treated mice showed less perivascular edema than the untreated group (Fig. 4d and f). At 48 h p.i., a distinct alveolar edema formation was additionally observed, which was diminished in VT-treated mice in contrast to PBS-treated mice (Fig. 4e and g). Notably, pulmonary leukocyte recruitment and pulmonary inflammatory cytokine release were not significantly affected by VT treatment (Fig. 5a–d, Additional file 1: Figure S4). Only IL-12p40 levels were reduced in BALF 48 h after infection (Fig. 5d). Furthermore, systemic inflammatory responses at 24 h and 48 h p.i. remained unaffected by VT treatment, as indicated by quantification of blood leukocytes (Fig. 6a and b) as well as IL-6 and KC plasma levels (Fig. 6c and d). The latter were slightly decreased by trend upon VT treatment, with only IL-6 levels at 24 h p.i. significantly reduced. Furthermore, bacterial burden in BALF (Additional file 1: Figure S5A and B) and blood (Additional file 1: Figure S5C and D) was not significantly reduced by VT treatment in contrast to the PBS-treated groups at both time points. In vitro testing failed to show a direct antimicrobial activity of VT (Additional file 1: Figure S6).

## Discussion

Despite potent antibiotic treatment, pathogen-host interaction in severe pneumonia may evoke an increase in pulmonary endothelial permeability [44], resulting in life-threatening acute lung injury [45]. Virulence factors of the pathogens, including specific bacterial toxins as well as an uncontrolled host immune response, may induce lung barrier dysfunction [2]. Therapeutic stabilization of the pulmonary endothelial barrier is crucial to reducing acute lung injury and lung edema in severe pneumonia [11]. The present study provides evidence that the novel Tie2 agonist VT promotes pulmonary barrier function in pneumococcal pneumonia-induced lung injury. This synthetic peptide is chemically stable [23–25] and was reported to improve endothelial barrier function in different in vitro and in vivo experimental models [25–28].

The present study shows, for the first time to our knowledge, that VT directly binds to the murine Tie2 receptor with a dissociation constant in the low nanomolar range. This observation correlates with the dose-dependent phosphorylation of Tie2 following application of VT to mouse lung microvascular endothelial cells. The latter result confirms findings of other studies in which researchers have investigated other cell types/tissues [24–26, 28, 33].

In vitro we observed that VT protected hPMVEC from PLY-induced damage. PLY is a hemolytic exotoxin and an important virulence factor of *S. pneumoniae* [29, 30], the most frequent pathogen in CAP [3, 5]. This protective effect of VT is in accordance with results reported by David et al. [25], who showed that VT treatment protects hPMVEC after LPS stimulation.

To expand on these first results, we tested effects of VT in mouse models of pneumonia of different complexity (in vitro, ex vivo, and in vivo). Again, pretreatment with VT protected from PLY-induced pulmonary hyperpermeability in an in vitro model of isolated mLEC and in the more complex ex vivo model of isolated perfused and ventilated mouse lungs. These results are consistent with findings of other groups showing a protective effect of VT in murine endotoxemia and abdominal sepsis [25, 26], in acute skin ionizing radiation in mice [33], and in experimental acute kidney injury [27].

Next, we tested whether VT is capable of protecting from pneumonia-induced lung injury in vivo. Using a murine model of pneumococcal pneumonia, we chose a clinically relevant therapeutic approach and treated mice intravenously twice daily with VT, beginning when pneumonia had already been established (22 h p.i.) [35, 43, 46]. In this experimental in vivo setting, VT was shown to quickly and effectively reduce pulmonary hyperpermeability in a dose-dependent manner. Two hours after the first VT application, and likewise 48 h p.i. after threefold VT application, the HSA BALF/plasma ratio was decreased. To test for further effects of VT in pneumonia other than barrier stabilization, we performed a second set of in vivo experiments using VT at the highest dose (500 ng). We could reproduce the significant protective effect of VT on pneumonia-induced hyperpermeability and confirm these findings with histological analyses of lungs. The distinct perivascular edema formation was reduced in the VT-treated group 24 h and 48 h p.i. Furthermore, lungs of VT-treated mice were nearly devoid of the additional massive alveolar edema formation observed in the infected PBS-treated group 48 h p.i. This protection did not seem to be caused by a decrease in the inflammatory response. Pulmonary recruitment of immune cells, cytokine release, and the systemic immune response at both investigated time points (24 h and 48 h p.i.) was only slightly affected by VT treatment. Furthermore, no significant effect of VT on bacterial burden in lungs and blood was seen. These results are consistent with the observation by Sugiyama et al. [28], who showed a protective effect of VT on pulmonary barrier function in a murine model of influenza pneumonia without affecting leukocyte influx into the lung or repressing virus replication. However, the results differ from the observations of Kümpers et al. [26], who showed reductions in local and systemic inflammatory markers after abdominal sepsis. A possible explanation for the seemingly contradictory findings is the use of different treatment protocols. In comparison to the work of Kümpers et al., who started the treatment at the beginning of peritoneal sepsis (2 h after cecal ligation and puncture), we started at a rather late time point when severe pneumonia was established and exacerbated into acute lung injury and the recruitment of leukocytes was already near the maximum. It cannot be excluded that VT given in an earlier stage of the disease might decrease pulmonary inflammation. Further studies are necessary to investigate whether VT is generally capable of influencing the pulmonary immune response in pneumonia.

In our study, only the cytokine IL-12p40 was decreased in BALF 48 h after infection, and the proinflammatory cytokine IL-6 concentration in blood was reduced after 24 h, whereas levels of all other analyzed cytokines did not differ between groups. These results could indicate an influence of VT on specific immune response mechanisms. It cannot be excluded that VT directly binds to the Tie2 receptor expressed on immune cells as described for monocytes/macrophages in angiogenesis [47–49] or in chronically asthmatic mice [50], rather than affecting, for example, mechanisms of cell recruitment into the lungs. However, most of our findings suggest that VT primarily exerts a direct impact on the pulmonary endothelial barrier. This assumption is supported by our findings from studies of endothelial cells and isolated perfused and ventilated lungs, which both are devoid of recruited immune cells [51].

## Conclusions

In summary, VT was shown to protect pulmonary barrier function without relevantly diminishing the immune response. Of note, immune cell influx into the lungs was unaffected by VT treatment, thereby ensuring effective bacterial elimination. Furthermore, an intact immune system is also crucial to avoiding secondary infections. The results of our study, together with characteristics of VT, such as receptor specificity, chemical stability, and low production costs [28], suggest that VT may possess great potential as a novel therapeutic agent for reduction of permeability in pneumococcal pneumonia-induced lung injury.

## Additional file

Additional file 1: Figures S1–S6. Supplementary figures. (PDF 1144 kb)

## Abbreviations

Ang: Angiopoietin
BAL: Bronchoalveolar lavage
BALF: Bronchoalveolar lavage fluid
CAP: Community-acquired pneumonia
CFU: Colony-forming units
ECIS: Electric cell-substrate impedance sensing
ELISA: Enzyme-linked immunosorbent assay
hPMVEC: Human pulmonary microvascular endothelial cells
HSA: Human serum albumin
IgG: Immunoglobulin G
IL: Interleukin
LPS: Lipopolysaccharide
mLEC: Murine lung endothelial cells
p.i.: post infection
PLY: Pneumolysin
PMN: Polymorphonuclear cells
RLU: Relative light units
TER: Transcellular electrical resistance
VE: Vascular endothelial
VT: Vasculotide
