# Supplementary material for: Vasculotide reduces pulmonary hyperpermeability in experimental pneumococcal pneumonia
Source: Crit Care. 2017 Nov 13;21:274. doi: 10.1186/s13054-017-1851-6 (PMC5683375; doi:10.1186/s13054-017-1851-6)

Supplementary Figures

Figure S1. Experimental design for *in vivo* study. Mice were infected with *S. pneumoniae* (*S. pn.*; 5x10^6 CFU/mouse) or sham infected with PBS (20 µl) and intravenously (i.v.) treated with different dosages of Vasculotide (VT; 100 ng, 200 ng or 500 ng) or PBS 22 h, 34 h and 46 h post infection (p.i.). 24 h or 48 h p.i. mice were anesthetized and different analyses performed.

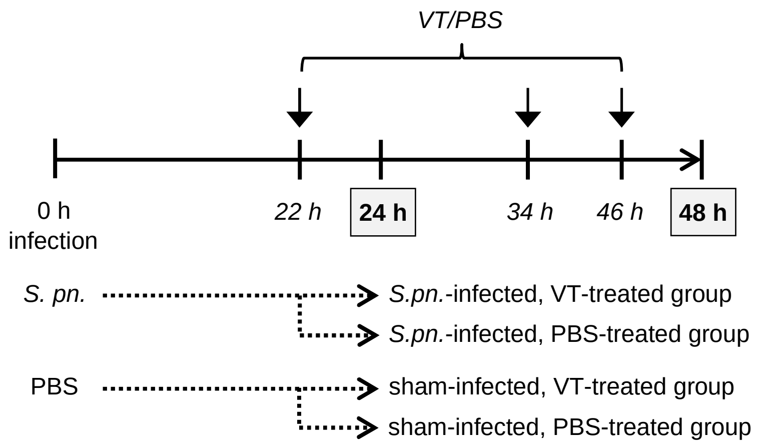

Figure S2. Vasculotide therapy did not affect clinical outcome in *S. pneumoniae* infected mice. *S. pneumoniae* infected mice (*S. pn.*, 5x10^6 CFU/mouse) or sham-infected mice were intravenously (i.v.) treated with Vasculotide (VT; 500 ng) or PBS and body weight and body temperature were measured 22 h, 34 h and 46 h post infection (p.i.). VT did not influence body weight (A) and body temperature (B) 46 h p.i. in comparison to infected, PBS-treated mice. Values are given as mean + SEM (A) or mean ± SEM (B). n = 8 (*S. pneumoniae* infected groups) or 5 (sham-infected groups).

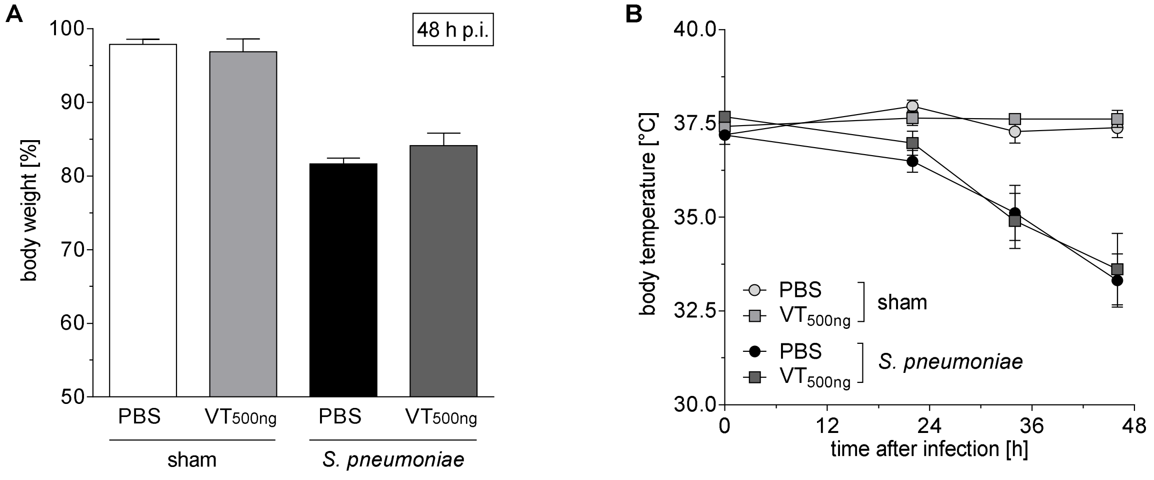

Figure S3. Reduced lung injury in Vasculotide-treated mice. *S. pneumoniae* (*S. pn.*, 5x10^{6} CFU/mouse) or sham-infected mice were intravenously (i.v.) treated with Vasculotide (VT; 500 ng) or PBS 22 h, 34 h and 46 h post infection (p.i.). For histological analysis, lungs were prepared and fixed 24 h or 48 h p.i. Histological analysis revealed reduced lung injury in VT-treated mice as compared to solvent (PBS)-treated animals 24 h and 48 h p.i. Representative images are shown (n = 3-4), *bar* 2 mm.

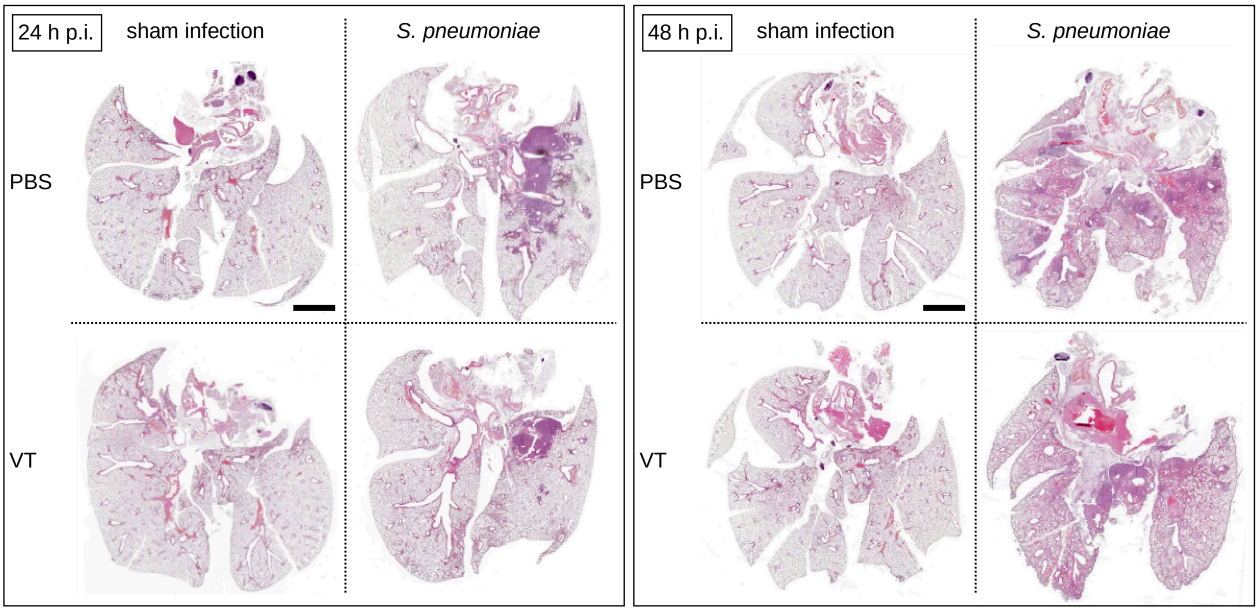

Figure S4. Vasculotide did not significantly reduce pulmonary leukocyte recruitment. *S. pneumoniae* (*S. pn.*, 5x10^6 CFU/mouse) or sham-infected mice were intravenously (i.v.) treated with Vasculotide (VT; 500 ng) or PBS 22 h, 34 h and 46 h post infection (p.i.). Lungs were prepared and bronchoalveolar lavage (BAL) was performed 24 h p.i. (A) or 48 h p.i (B). Leukocytes in BAL fluid (BALF) were differentiated by FACS analysis. Values are given as mean + SEM. n = 8 (*S. pneumoniae* infected groups) or 5 (sham-infected groups).

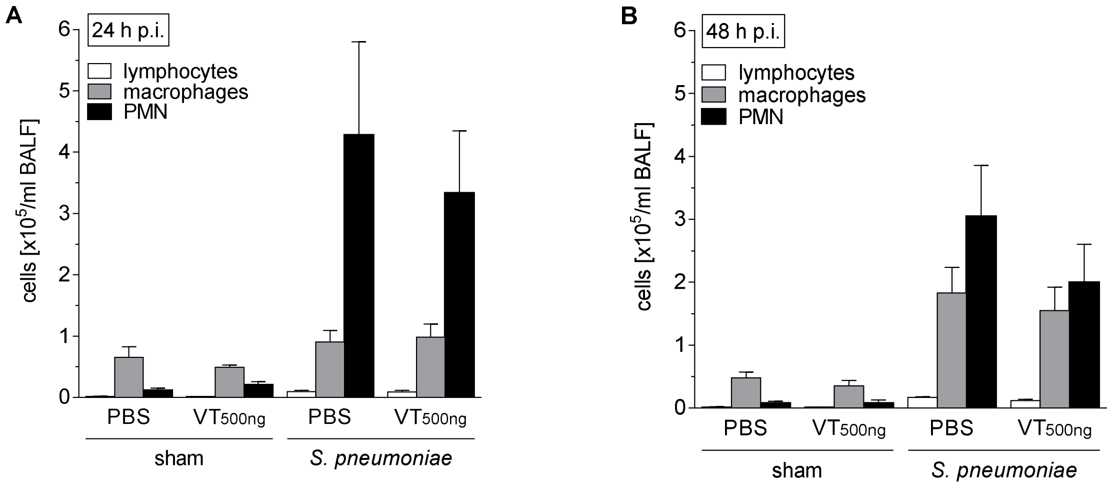

Figure S5. Vasculotide therapy did not significantly influence bacterial burden in lungs and blood in *S. pneumoniae* infected mice. *S. pneumoniae* (*S. pn.*) infected mice ($5x10^6$ CFU/mouse) were intravenously (i.v.) treated with Vasculotide (VT; 500 ng) or PBS 22 h, 34 h and 46 h post infection (p.i.). The bacterial burden was quantified in bronchoalveolar lavage fluid (BALF) and in blood 24 h or 48 h p.i. VT therapy did not affect the pulmonary (A, B) or systemic (C, D) bacterial burden 24 h (A, C) and 48 h (B, D) after infection. Values are given as individual data and mean ± SEM (n = 8).

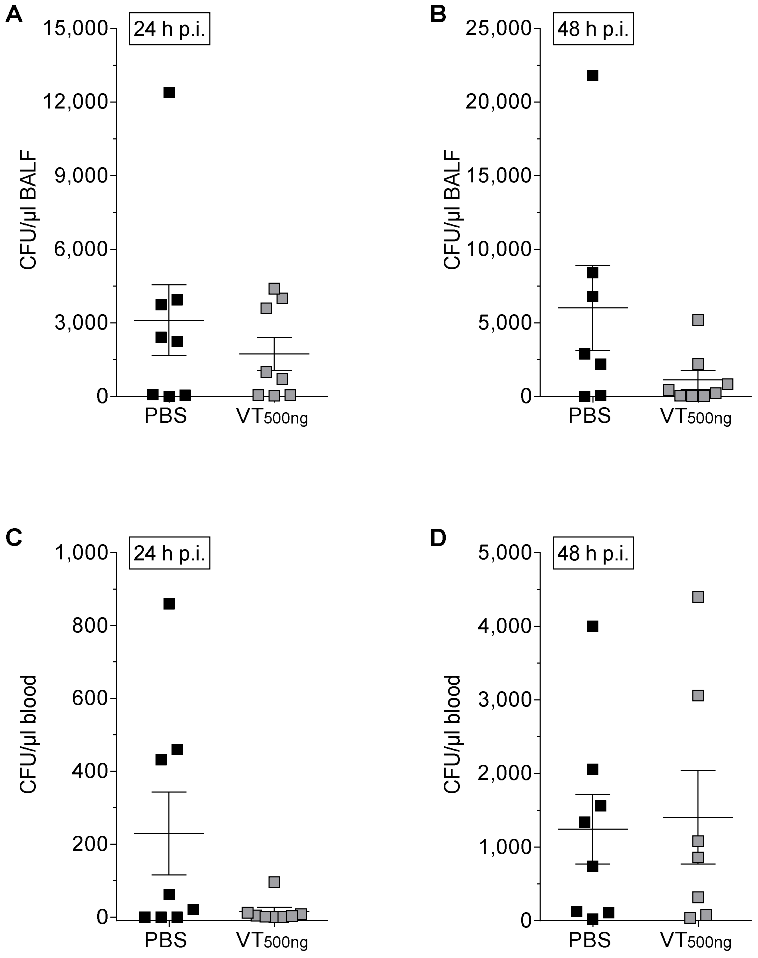

Figure S6. Vasculotide did not affect bacterial growth *in vitro*. In a preliminary experiment, we investigated if Vasculotide (VT) has a direct effect on the bacterial growth of *Streptococcus pneumoniae*. Capsulated *S. pneumoniae* (*S. pn.*) serotype 3 (NCTC7978) was cultured on Columbia blood agar + 5% sheep blood for 8 h. Single colonies were transferred into THY media to yield OD_{600} = 0.03-0.04. Liquid cultures were incubated at 37°C and 5% CO_2 for 2.5-4 h until the bacteria reached a phase of logarithmic growth (OD_{600} = 0.3-0.4). 30 min later, log-phase bacterial cultures were treated with VT (5 µg/ml) or PBS. Optical density was measured every 30 min for further 5 hours. Individual time-proliferation curves are shown.

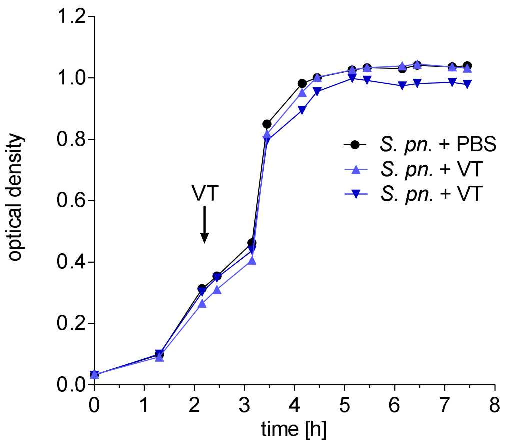

Supplement: Additional file 1: Figures S1–S6. — Supplementary figures. (PDF 1144 kb) [file 13054_2017_1851_MOESM1_ESM.pdf]
